# Experimental investigation of the susceptibility of Italian *Culex pipiens* mosquitoes to Zika virus infection

**DOI:** 10.2807/1560-7917.ES.2016.21.35.30328

**Published:** 2016-09-01

**Authors:** Daniela Boccolini, Luciano Toma, Marco Di Luca, Francesco Severini, R Romi, Maria Elena Remoli, Michela Sabbatucci, Giulietta Venturi, Giovanni Rezza, Claudia Fortuna

**Affiliations:** 1 Department of Infectious Diseases, Istituto Superiore di Sanità, Rome, Italy; 2 These authors contributed equally to this manuscript; 3 European Programme for Public Health Microbiology Training (EUPHEM), European Centre for Disease Prevention and Control (ECDC), Stockholm, Sweden

**Keywords:** Zika virus, Italy, *Cx pipiens*, vector competence, *Ae aegypti*, experimental infection

## Abstract

We investigated the susceptibility of an Italian population of *Culex pipiens* mosquitoes to Zika virus (ZIKV) infection, tested in parallel with *Aedes aegypti*, as a positive control. We analysed mosquitoes at 0, 3, 7, 10, 14, 20 and 24 days after an infectious blood meal. Viral RNA was detected in the body of *Cx. pipiens* up to three days post-infection, but not at later time points. Our results indicate that *Cx. pipiens* is not susceptible to ZIKV infection.

Since its emergence in South and Central America in 2014, Zika virus (ZIKV) has spread rapidly, resulting in an unprecedentedly large number of infections [1-4]. It is well accepted that *Aedes* species are the main vectors of ZIKV [5-7]. However, in order to assess the risk of spread of this infection to new areas, it is pivotal to investigate the possibility that mosquito species belonging to other genera could contribute to sustaining virus transmission. *Culex pipiens* is widespread in Mediterranean countries [8], and little is known at present about its potential role as ZIKV vector. We report here our findings on experimental infection of an Italian population of *Culex pipiens* mosquitoes with ZIKV, using *Ae. aegypti* mosquitoes as a positive control. Using quantitative reverse transcription PCR (qRT-PCR) to detect viral RNA, our findings indicate that *Cx. pipiens* is not susceptible to ZIKV infection.

## Experimental infection of mosquitoes

Experimental infection of the mosquitoes, starting in April 2016, was performed using the ZIKV H/PF/2013 strain, of the Asian genotype (kindly provided by Dr Isabelle Leparc-Goffart of the French National Reference Centre for Arboviruses in Marseille) isolated from a patient returning from French Polynesia in 2013 [9]. We exposed 10 day-old female mosquitoes from an Italian *Cx. pipiens* population (collected in Rome, Latium Region, in the summer of 2015) and from a long-established colony of *Ae. aegypti* (collected in Reynosa, Mexico, in 1998) to an infectious blood meal for one hour, through a membrane feeding apparatus.

The virus was diluted in rabbit blood (final virus concentration: 6.46 log_10_ plaque-forming units (PFU)/mL) and maintained at 37 °C by a warm-water circulation system. After the blood meal, fully engorged females were transferred to other cages and maintained on a 10% sucrose solution in a climatic chamber (26 ± 1 °C; 70% relative humidity; 14 hour light:10 hour dark cycle) for 24 days. A total of 8–10 mosquitoes from both species were processed individually at 0, 3, 7, 10, 14, 20 and 24 dpi.

To evaluate viral infection, dissemination and transmission, body (head, thorax and abdomen), legs plus wings, and saliva were analysed, as previously described [10]. The viral titre was evaluated by qRT-PCR. Specific primers ZIKV 1086 and ZIKV 1162c were used, with 5-FAM as the reporter dye for the probe (ZIKV 1107-FAM) [11]. Crossing point values were compared with a standard curve obtained from 10-fold serial dilutions of virus stock of known concentration [7]. 

Mosquito bodies were analysed in order to evaluate the infection rate, calculated as the number of ZIKV-positive mosquito bodies out of the total number of fed females. Legs plus wings were tested to assess the dissemination rate, calculated as the number of the specimens with ZIKV-positive legs plus wings among the tested mosquitoes. The saliva of the potentially infected females was processed to assess the transmission rate, defined as the number of mosquitoes with ZIKV-positive saliva among the number of tested mosquitoes [7,10].

## Vector competence analysis

All the *Cx. pipiens* (n=10) and *Ae. aegypti* (n=8) bodies analysed at day 0 (i.e. immediately after the infectious blood meal) showed positive results, with mean viral titres of 4.23 (standard deviation (SD): 0.07) log_10_ PFU/mL and 3.7 (SD: 0.18) log_10_ PFU/mL, respectively, confirming the ingestion of viral particles.

At 3 dpi, only one of 10 *Cx. pipiens* mosquitoes analysed was infected. In the *Cx. pipiens* body, viral RNA was detected at a low concentration (0.17 log_10_ PFU/mL), whereas no viral RNA was detected at the later collection times. Viral RNA was never detected in legs plus wings and in the saliva of the *Cx. pipiens* (Table).

**Table:** Competence for Zika virus (infection, dissemination and transmission rates)^a^ and Zika virus titres in body, legs plus wings and saliva of *Culex* *pipiens* and *Aedes aegypti* colonies fed orally^b,c^

Days post-infection	*Cx. pipiens*						*Ae. aegypti*					
	Infection rate	Mean viral titre^d^ (SD) in body	Dissemination rate	Mean viral titre^d^ in legs + wings	Transmission rate	Mean viral titre^d^ in saliva	Infection rate	Mean viral titre^d^ (SD) in body	Dissemination rate	Mean viral titre^d^ (SD) in legs + wings	Transmission rate	Mean viral titre^d^ (SD) in saliva
0^e^	10/10	4.23 (0.07)	0/10	0	0/10	0	8/8	3.73 (0.18)	0/8	0	0/8	0
3	1/10	0.17	0/10	0	0/10	0	ND	ND	ND	ND	ND	ND
7	0/10	0	0/10	0	0/10	0	6/12	3.76 (1.25)	6/12	2.57 (0.32)	2/12	1.80 (0.14)
10	0/10	0	0/10	0	0/10	0	ND	ND	ND	ND	ND	ND
14	0/10	0	0/10	0	0/10	0	4/8	5.12 (0.06)	4/8	3.11 (0.36)	3/8	2.05 (0.97)
20	0/10	0	0/10	0	0/10	0	4/10	4.60 (0.21)	3/10	3.08 (0.28)	3/10	2.10 (0.39)
24	0/10	0	0/10	0	0/10	0	ND	ND	ND	ND	ND	ND

ND: not detected; SD: standard deviation.

^a^ Infection rate: number of virus-positive bodies/number of tested females; dissemination rate: number of virus-positive legs plus wings/number of tested females; transmission rate: number of virus-positive saliva samples/number of tested females.

^b^ The mosquitoes were kept at 26 °C and collected at various days post-infection.

^c^ The viral titre was evaluated by quantitative reverse transcription PCR (qRT-PCR). Crossing point values were compared with a standard curve obtained from 10-fold serial dilutions of virus stock of known concentration [7].

^d^ Expressed as log_10_ plaque-forming units/mL.

^e^ Immediately after the infectious blood meal.

These findings differed greatly with those obtained with *Ae. aegypt*i*.* As expected, in *Ae. aegypti*, the viral titres detected in the mosquito bodies increased gradually, reaching a mean value of 5.12 (SD: 0.06) log_10_ PFU/mL at 14 dpi, as well as in legs plus wings and in the saliva, showing an extrinsic incubation period similar to that previously described [7]. The infection rate at 7 dpi was 6/12 as was found for the dissemination rate. At the same collection time, ZIKV was detected also in the saliva with a transmission rate of 2/12 and a mean viral titre of 1.80 (SD: 0.14) log_10_ PFU/mL. In the later collection points, ZIKV was detected in body, legs plus wings and saliva confirming the expected vector competence of this mosquito species (Table).

## Discussion

In countries where ZIKV has recently spread, *Ae. aegypti* and *Ae. albopictus* have been recognised as the most efficient vectors [5-7]. There is limited evidence that ZIKV can infect other mosquito species naturally: the presence of the virus has been reported in species of the *Culex* genus in Senegal and in Brazil [12,13]. Following our study on ZIKV competence of an Italian *Ae. albopictus* population [7], we investigated the susceptibility of an Italian population of the widespread indigenous species *Cx. pipiens* [8] to ZIKV infection under laboratory conditions. Increasing concern about the spread of ZIKV and its epidemic potential [1-4] makes it particularly important to fill gaps in knowledge about the role that mosquitoes other than *Ae. albopictus* and *Ae. aegypti* may have in the circulation and transmission of this virus in the Mediterranean area.

We focused our attention on *Cx. pipiens* mosquitoes as a potential ZIKV vector, since these mosquitoes are ubiquitous in temperate and tropical areas, where they are involved in the transmission of a range of human and zoonotic pathogens, such as West Nile virus, St Louis encephalitis virus, Rift Valley Fever virus, filarial worms and avian malaria [14,15]. The important vector role of *Cx. pipiens* arises from its opportunistic host feeding behaviour and on the high abundance it can reach in rural as well as in urban settings [14,15].

Our results show that the Italian *Cx. pipiens* population tested was not susceptible to ZIKV; the short persistence of the virus in the mosquito’s body does not allow viral replication and, consequently, viral dissemination in the salivary glands. Conversely, our results showed *Ae. aegypti* to be competent for ZIKV transmission, as previously reported [7].

Similar results were reported in a recent study on ZIKV susceptibility of a *Cx. pipiens* population from the United States [16], showing that this species is not a competent vector for ZIKV. However, in Brazil, current studies have reported ZIKV detection in the salivary glands of *Cx. quinquefasciatus* that were artificially fed with ZIKV-infected blood, and tested 7 and 15 days post-feeding [13,17].

We did not carry out viral titration by plaque formation as we observed in a previous study a high correlation between titration by this method and viral RNA detection [10]: this may constitute a limitation of this study.

In conclusion, the findings of the studies conducted on Italian and United States populations of *Cx. pipiens* mosquitoes have important public health implications, and help to optimise the vector control activities in Italy, should autochthonous ZIKV transmission occur. *Cx. pipiens* mosquito populations in Italy are unlikely to be competent vectors for ZIKV. Thus, to date, *Ae. albopictus* is the only mosquito established in Italy for which vector competence for ZIKV has been demonstrated [7]. However, even if a low epidemic potential risk of ZIKV in Italy was estimated [18], it should be considered that arboviruses have the potential to rapidly change their vector–host associations [19]. Therefore further vector competence studies should be undertaken in order to plan evidence-based interventions.
